# The variability of physical enjoyment, physiological responses, and technical-tactical performance according to the bout duration of small-sided games: a comparative study between female and male soccer players

**DOI:** 10.1186/s13102-023-00794-1

**Published:** 2024-04-03

**Authors:** Zouhaier Farhani, Sofiene Amara, Mohamed Ben Aissa, Noomen Guelmami, Anissa Bouassida, Ismail Dergaa

**Affiliations:** 1 Sport Sciences, Health and Movement (3SM), University of Jendouba, Higher Institute of Sport and Physical Education of Kef, Kef, Tunisia; 2 High Institute of Sport and Physical Education of Ksar-Said University of Manouba, Tunis, Tunisia; 3 Research Unit (UR17JS01) Sports Performance, Health & Society, Higher Institute of Sport and Physical Education of Ksar Saîd, Universite de la Manouba, Tunis, 2010 Tunisia; 4 Postgraduate School of Public Health, Department of Health Sciences (DISSAL), University of Genoa, Genoa, Italy; 5 Primary Health Care Corporation (PHCC), P.O. Box 26555, Doha, Qatar; 6 Research Unit Physical Activity, Sport, and Health, UR18JS01, National Observatory of Sport, Tunis, 1003 Tunisia; 7 High Institute of Sport and Physical Education, University of Sfax, Sfax, Tunisia

**Keywords:** Physical activity enjoyment, Technical skills. Heart rate, Four-a-side

## Abstract

**Background:**

This study aimed to compare various factors, namely perceived enjoyment (PE), percentage of peak heart rate (%HRpeak), blood lactate (La), rating of perceived exertion (RPE), and technical-tactical performance among soccer players across different bout durations (CB: continuous bout, MIB: medium intermittent bouts, and SIB: short intermittent bouts) and between male and female players during four-a-side (4vs4) small-sided games (SSGs) including goalkeepers.

**Methods:**

sixteen female soccer players (age: 20.1 ± 0.5 years old) and sixteen professional male adults (age: 20.7 ± 0.7 years old) participated in the study. SSGs (4vs4) were performed in a CB: (1 × 12 min), and in an interval format: MIB: (2 × 6 min), and SIB: (3 × 4 min) with 2 min of passive recovery. PE was collected after each SSGs. The players’ heart rate (HR) was continuously measured, whereas ratings of perceived exertion (RPE) and, blood lactate concentration ([La]) were determined at the end of each SSGs. Technical-tactical performance were analyzed during each session of SSGs. Pitch dimensions were (length x width) (25 × 32 m), and relative space per players was 100 m^2^.

**Results:**

For female soccer players, medium intermittent bouts (MIB) elicited significantly higher perceived enjoyment (PE) compared to continuous bouts (CB) (*p* < 0.001) and short intermittent bouts (SIB) (*p* < 0.01). Conversely, for male soccer players, CB resulted in higher PE compared to MIB (*p* < 0.001) and SIB (*p* < 0.001). During CB and MIB, peak heart rate (PeakHR) and percentage of peak heart rate (%HRpeak) were significantly higher in female players compared to SIB (PeakHR: CB: *p* < 0.001; PeakHR: MIB: *p* < 0.01; %HRpeak: CB: *p* < 0.001; %HRpeak: MIB: *p* < 0.01). Blood lactate (La) and rating of perceived exertion (RPE) were significantly greater in CB compared to MIB (La: *p* < 0.001; RPE: *p* < 0.01) and SIB (La: *p* < 0.001; RPE: *p* < 0.001) for female players only. For male players, CB resulted in significantly higher PeakHR, %HRpeak, La, and RPE compared to MIB (peak HR: *p* < 0.01, dunb = 1.35; %HR: *p* < 0.01; La: *p* < 0.01; RPE: *p* < 0.01) and SIB (peak HR: *p* < 0,01; %HR: *p* < 0.01; RPE: md = 0.87, *p* < 0.05). Regarding technical-tactical performance, in female players, the % of successful passes, successful tackles, and successful duels were higher during SIB compared to CB (*p* < 0.01; *p* < 0.001; *p* < 0.001) and MIB compared to CB (*p* < 0.01; *p* < 0.001; *p* < 0.001), while ball loss was lower during SIB compared to CB (*p* < 0.001) and MIB compared to CB (*p* < 0.001). In male players, % of successful passes and tackles were higher during CB compared to MIB (*p* < 0.001 and *p* < 0.05) and SIB (*p* < 0.001 and *p* < 0.05), while CB had a lower % of ball loss compared to MIB (*p* < 0.01) and SIB (*p* < 0.001). There was no significant difference in the % of successful duels between the bouts for either gender.

**Conclusion:**

This study showed a difference in physical enjoyment between male and female soccer players depending on the bout duration of SSGs. For that, trainers should consider intermittent bouts for female soccer players and continuous bouts for male soccer players when designing SSGs-based training in order to significantly improve PE, training load, and technical-tactical performance.

## Introduction

The scientific interest on small-sided games has been conducted using men and women soccer teams to quantify the physiological demands and physical exertion during training [1, 2]. Small-sided games (SSGs) are a form of soccer training specifically designed to simulate match conditions [1]. To achieve this, they are played on smaller fields with fewer players compared to traditional football matches and often involve modified rules Top of Form [3]. Small-sided games enable the simultaneous development of physical exertion, technical abilities, and tactical skills. [4, 5] without exposing players to the full physical load of a match [6] and they enable the simultaneous development of physical exertion, technical, and tactical skills [7, 8] than other types of training. Indeed intrinsic motivation of players induces more physical enjoyment during training based on SSGs [9]. For that, coaches and physical trainers use these spontaneous forms of specific training because: On one hand, they can manipulate the exercise intensity [10], on the other hand, they can encourage the mental improvement of players [11]. Furthermore SSGs are very widely used across the world and appreciated by players and trainers at the same time [9, 12]. Previous studies demonstrated that the length of the game and the number of players during SSGs induced different physiological and physical responses [13, 14], by modifying the duration and the number of high-intensity actions [15]. For this reason, it’s important to think about the causes which result performance decrease during SSGs. Some studies [16, 17] explained this decrease by peripheral and mental fatigue [9, 14], other studies thought about the psychological state of the players during training [18]. In the last reflection [9, 18] indicated that enjoyment affects pleasure to play and intrinsic motivation during SSGs. To the best of our knowledge, despite the wealth of studies on physical activity profiles and time-motion in male and female soccer during SSGs training [19–21], no study has attempted to compare the effects of SSGs bout duration on the perceived enjoyment, physiological responses and technical-tactical performances in female and male soccer players and no information regarding the comparison in physical enjoyment and work load during the bout duration between gender. The aim of the current study was to compare various factors, namely PE, %HRpeak, La, RPE, and technical-tactical performance among soccer players across different bout durations (CB, MIB, and SIB) and between male and female players during 4vs4 SSGs including goalkeepers. The findings from this study can be used to assist coaches to make decisions regarding the length of the bout that can be used during SSGs training in female and male soccer players in order to enhance the physical enjoyment and commitment of players to achieve training goals. In this study, we aimed to address this gap in the literature. Our initial hypothesis was that the length of bout duration affects physical enjoyment, increases physiological responses, and influences the technical-tactical performance in female soccer players more than male soccer players.

## Methods

### Participants

Two experimental groups included 16 male soccer players (age: 20.7 ± 0.7 years, height: 179.5 ± 6.1 cm, body mass: 67.2 ± 4.9 kg, body fat: 10.7 ± 0.7%) and 16 female soccer players (age: 20.1 ± 0.5 years, height: 165.5 ± 2.1 cm, body mass: 60 ± 3.5 kg, body fat: 12.3 ± 0.3%) voluntarily participated in this study. All players had a minimum 5 years of training experience and each group competed for the same team at a national league in Tunisia (high-level), and trained 5 times per week (7.5 ± 1.54 h of training/week), with one official competition. Goalkeepers were excluded from the analysis. All players were informed about the procedures of the study and the possible risks involved and signed a written informed consent form before participation. The study was approved by the Ethics Committee of the University of Jendouba (06/a-2023) and was consistent with the institutional ethical requirements for human experimentation in accordance with the Declaration of Helsinki as it was revised in 2013.

### Study design

This study was conducted during the middle of competitive season from (March to April 2022), prior to starting the study, players took part in a familiarization session to become accustomed to the testing procedures and gain experience with both the three SSGs bout duration into two consecutive weeks (2 days per week). At this time, anthropometric measures were also taken. Following familiarization, participants performed a Yo-Yo intermittent recovery test level 1 to record the individual maximal heart rate value (HRpeak) and participants was ranked according to the distance covered in this test. The coach also provided a subjective rating on the overall technical/tactical skill level for each player using a 5-point scale (from 1 = poor, to 5 = excellent) in an attempt to avoid skill and fitness mismatches and a subsequent imbalance in opposing SSGs teams. The SSGs protocol involved games with teams of 4vs4 (2 female teams and 2 male teams), on pitch sized 25 × 32 m. The SSGs’ net durations were performed either as (a) one bout of twelve minutes (1 × 12 min) or (b) two bouts of six minutes (2 × 6 min) or (c) three bouts of four minutes (3 × 4 min). The relative pitch size (m^2^/number of player) was 100. HR was continuously monitored at 5-s intervals throughout the SSGs. PE, La, and RPE were recorded at the end of each bout duration. SSGs was performed with the goalkeeper but was not included in the study. Supervision and verbal encouragement were continuously provided by the coaches [22, 23] and spare balls were available to each team whenever ball was out of bounds or when a goal was scored. The characteristics of the SSGs (number of bouts, bout duration [min], passive recovery duration (min), pitch dimensions [length x width]), recovery time and rest type are shown in Table 1. All measurements and SSGs sessions were performed at the same time (between 14.00 and 15.30 p.m.) on the same synthetic grass pitch during mid-season. A maximum of two sessions per week were undertaken so that coaches can enhance others technical and tactical skills for players during the same week. On Sundays, players played a match and Monday was dedicated to recovery activities. Each SSGs was preceded by a 20-min specific warm-up, which consisted of low-intensity running, striding, and stretching. Players were allowed to consume water ad-libitum during recovery.

### Anthropometric measurements

Measurements of body mass and height were performed using a wall-mounted stadiometer and an electronic scale, respectively. Biceps, triceps, subscapular, and suprailiac skinfolds were measured in mm using a standard calliper (Baty International, West Sussex, England). Body density and percentage of body fat were calculated using the following formulas [24].

Body density = 1.1765 − 0.0744*(log10 ΣS), where ΣS is the sum of the skinfolds.

Body fat (%) = (4.95/D − 4.50) *100, where D is body density.

### Yo-Yo intermittent recovery test

The Yo-Yo Intermittent Recovery test (level one) was used to measure aerobic performance and characterize the sample in this regard [25]. This protocol consists of repeated 20-m shuttle runs between the starting, turning, and finishing lines. The test continues at a progressively increasing speed which is controlled by audio bleeps from a recording. The speed level begins at 10 km/h. The test was stopped when a participant could no longer maintain the required running speed dictated by the bleep for two consecutive occasions, or if they felt that they could not complete the stage. The test was performed on a synthetic grass field in groups comprised of six players. The highest heart rate measurement (HRpeak) was recorded.

### Small-sided games (SSGs)

The SSGs involved games with teams of 4vs4 on pitches size 25 × 32 m. The relative pitch size (m2/number of players) was 100. The goals dimensions were 3 m in length and 2 m in height. Three different SSGs net durations were (a) one bout of twelve minutes (1 × 12 min), (b) two bouts of six minutes (2 × 6 min), and (c) three bouts of four minutes (3 × 4 min). HR was continuously monitored in five-second intervals throughout the SSGs. Perceived enjoyment and rate of perceived exertion (RPE) were recorded at the end of each game. Passive recovery of two minutes was allowed between the intermittent SSGs bouts.

### Physical activity enjoyment scale (PACES)

Enjoyment of physical activity was assessed by the 18-item Physical Activity Enjoyment Scale (PACES) [26]. All participants rated how they “feel at the moment about the physical activity you have just been doing”, using a 7-point scale (e.g., 1 = “It’s very pleasant”; 7 = “It’s not fun at all”). Overall enjoyment of physical activity means score was generated by summing the individual item means scores, yielding a potential score range from 18 to 126.

### Heart rate measurement

Heart rate was monitored using a Polar Team Sports System (Polar Electro Oy, Kempele, Finland). Stored data were transferred to a computer and filtered by Polar Precision Performance Software 4.0 (Polar Electro Oy, Kempele, Finland). The percentage of the peak heart rate (%HRpeak) attained in each SSGs was used to assess cardiovascular acute adaptations to exercise. %HRpeak was calculated by dividing the mean heart rate of the SSG by the peak heart rate times 100; %HRpeak = (HRgame/HRpeak)*100 [27]. The mean heart rate of each SSG was calculated averaging the mean heart rate of each bout and the peak heart rate was obtained in the Yo-Yo test.

### Blood lactate

Blood lactate (La) was measured during the 3–4 min after the end of the last bout of each SSGs as shown in the study of [28]. The blood samples were collected at the fingertip of the index finger and were analysed by a portable lactate monitor (Lactate Pro, Arkray, Japan) who has been previously validated [29].

### Rate of perceived exertion (RPE)

After the last bout of each SSGs format, the perceived intensity of the training sessions for each player was evaluated using the Borg CR-10 Scale for perceived exertion as proposed by [30]. Players were familiarized with the CR-10 scale. This scale has been validated as an indicator of training intensity in intermittent studies using SSGs in soccer [31].

### Statistical analysis

Data are presented as means ± SD. The Kolmogorov-Smirnov test and Levene’s statistic were used to verify the normality of the data and the homogeneity of variance. A Mixed Analysis of Variance (ANOVA) with repeated measurements, applying a factorial design of [3 bouts duration] x [2 genders], was executed to assess the significance of differences among Small-Sided Games (SSGs) on each dependent variable. Additionally, a three-way ANOVA incorporating [3 bouts duration] x [2 Times] x [2 genders] was conducted to examine differences in Profile of Mood States (POOMS) scores.

A Bonferroni Post-Hoc test was applied to make a pairwise comparison between the three SSGs format (i.e.,12, 2 × 6 and 3 × 4 min). Confidence intervals (95% CI) were calculated to indicate the magnitude of change. Effect size was evaluated with eta partial squared (ηp2), where 0.01 < ηp2 < 0.06 constitutes a small effect, 0.06 ≤ ηp2 ≤ 0.14 constitutes a medium effect, and ηp2 > 0.14 constitutes a large effect [32]. Where significant differences were detected, post-hoc pairwise comparisons, also with Bonferroni corrections, identified where these differences occurred. The magnitude of the differences for the post-hoc comparisons was calculated as Cohen’s d with Hedges’ corrections [32]. This value is reported as unbiased Cohen’s d (dunb) [33], with dunb < 0.50, constituting a small effect, 0.50 ≤ dunb ≤ 0.79 moderate, and dunb ≥ 0.80 a large effect [34]. Hereunder, dunb will be named as d.95% confidence intervals (95%CI) for the differences were also calculated [35, 36]. Data were analyzed with the statistical software SPSS 28.0. The level of significance was set at *p* < 0.05.

## Results

### Perceived enjoyment

Regarding the perceived enjoyment, Mixed ANOVA testing showed a significant interaction effect between both factors: bout duration x gender (F_2,30_ = 294.42, *p* < 0.001, ηp 2 = 0.9). Indeed, among female soccer players, PE scores are inversely proportional to the length of the bout duration of SSGs. Bonferroni post hoc analyses showed that there was a lower PE for continuous bout duration 1 × 12 min compared to the medium intermittent bout duration 2 × 6 min [mean difference (md) = 15.75, *p* < 0.001, d_unb_ = 6.74, 95%CI = 13.43 to 18.06] and the short intermittent bout duration 3 × 4 min [md = 14.37, *p* < 0.01, d_umb_ = 4.93, 95%CI = 11.57 to 17.14]. Similarly, the PE scores are significantly lower in the medium intermittent bout duration (2 × 6 min) than that of the short intermittent bout duration (3 × 4 min): [md = 1.37, *p* < 0.05, d_umb_ = 0.57, 95%CI = 0.51 to 2.49].

**Fig. 1:**
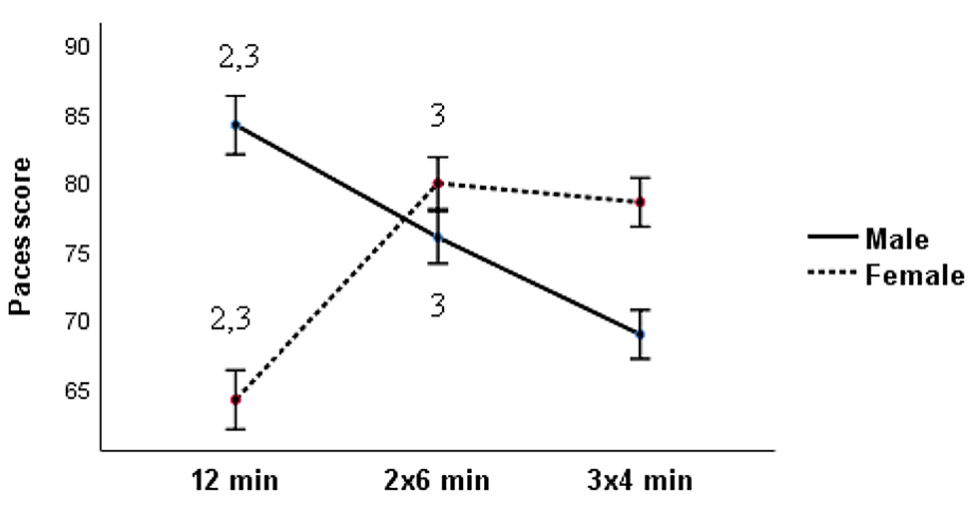
Comparison between means paces score of male and female soccer players after different bout durations of small-sided games (12 min, 2 × 6 min, and 3 × 4 min). 1,2,3: statistically significant difference within Condition 1: 1 × 12 min; 2: 2 × 6 min; 3: 3 × 4 min respectively. PACES: Physical Activity Enjoyment Scale

On the other hand, among male soccer players, continuous bout duration (1 × 12) was characterized by a significantly higher PE (Fig. 1) compared to medium intermittent bout duration 2 × 6 min (md = 8.18 *p* < 0.001, 95%CI [6.18–10.18], d_umb_ = 1.62 and the short intermittent bout duration 3 × 4 min (md = 15.25, *p* < 0.001, 95%CI [12.14–18.35], d_umb_ = 3.33. The same, our results showed also significantly higher PE scores during the medium intermittent bout duration (2 × 6 min) compared to the short intermittent bout duration 3 × 4 min (m.d = 7.06, *p* < 0.001, 95%CI [4.15–9.96], d_umb_ = 1.59). The full set of post-hoc pairwise comparisons and interaction effects are presented in Fig. 1.

### Physiological responses

Statistical analysis showed a significant interaction effect between bout duration and gender on the peak HR of the game: F (_2.30_) = 5.27, *p* < 0.01, ηp^2^ = 0.15, % HR peak: F(_2.30_) = 5.38, *p* < 0.01, ηp^2^ = 0.15, La: F(_2,30_) = 21.69, *p* < 0.001, ηp2 = 0.4 and RPE: F(_2,30_) = 4.9, *p* < 0.05, ηp^2^ = 0.14 (Table 1). Bonferroni post hoc analyses showed significant differences within bout duration in female as well as in male soccer players. In effect, during continuous bout duration 1 × 12 min and medium intermittent bout duration 2 × 6 min, PeakHR and %HRpeak was significantly increased in female soccer players compared to the short intermittent bout duration 3 × 4 min [PeakHR: 1 × 2 min: md = 5, *p* < 0.001,d_unb_ = 2.18, 95%CI = 3.29 to 6.7, PeakHR: 2 × 6 min: md = 4.31, *p* < 0.01, d_unb_ = 0.73, 95%CI = 1.39 to 7.23], [%HRpeak: 1 × 12 min: md = 2.64, *p* < 0.001, d_unb_ = 1.85, 95%CI = 1.74 to 3.55, %HRpeak: 2 × 6 min: md = 2.28, *p* < 0.01, d_unb_ = 1.26, 95%CI = 0.74 to 3.82]. The values of La concentration and RPE were significantly greater only in continuous bout duration 1 × 12 min compared to the medium intermittent bout duration 2 × 6 min [La: md = 1.14, *p* < 0.001, d_unb_ = 2.59, 95%CI = 0.72 to 1.55; RPE: md = 1.18, *p* < 0.01, 95%CI = 0.44 to 1.93, d_unb_ = 1.66] and 3 × 4 min [La: md = 1.98, *p* < 0.001, d_unb_ =, 95%CI = 1.47 to 2.48; RPE: md = 1.62, *p* < 0.001, 95%CI = 0.93 to 31, d_unb_ = 2.02).

**Fig. 2:**
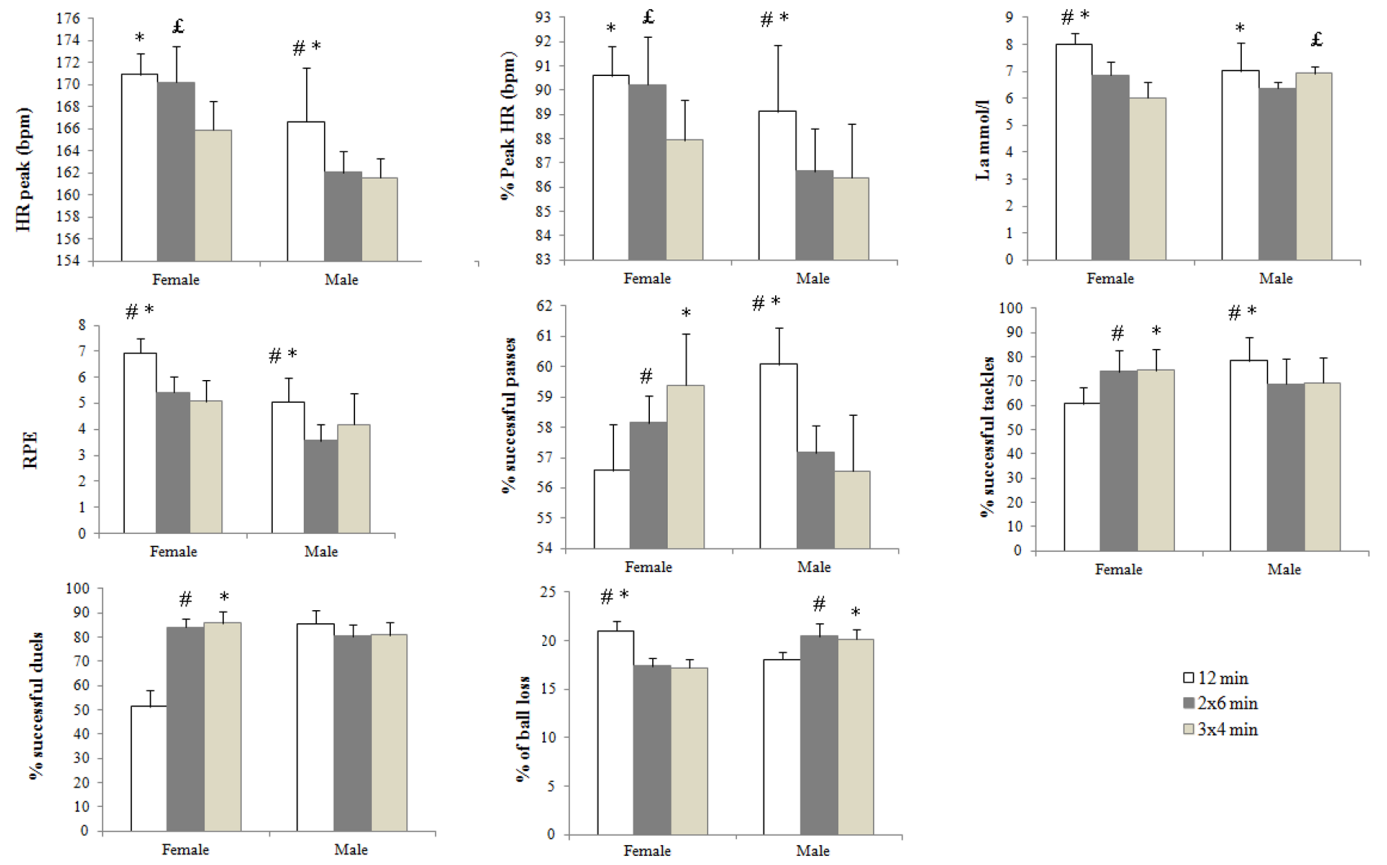
Mean values ± SD of physiological responses, RPE, and, technical-tactical performance, during 4vs4 soccer small-sided games (SSGs) at different bout duration (1 × 12 min, 2 × 6 min, and 3 × 4 min) for male and female soccer players. *: statistically significant difference between 1 × 12 min and 3 × 4 min; £: statistically significant difference between 2 × 6 min and 3 × 4 min; #: statistically significant difference between 1 × 12 min and 2 × 6 min. PACES: Physical Activity Enjoyment Scale; % HRpeak: percentage of the peak heart rate achieved; RPE: rate of perceived exertion measured with the Borg CR-10 scale; La: lactate

On the other hand, in male soccer players, Continuous bout duration “1 × 12 min” was characterized by a significantly greater peakHR, %HRpeak, La, and RPE compared to medium intermittent bout duration “2 × 6 min” [peak HR: md = 4.62, *p* < 0.01, d_unb_ = 1.35, 95%CI = 1.65 to 7.59; %HR: md = 2.46, *p* < 0.01, d_unb_ = 1.2, 95%CI = 0.89 to 4.03; La: md = 0.63 d_unb_ = 1.04, *p* < 0.01, 95%CI = -0.14 to 1.41; RPE: md = 1.5, *p* < 0.01, d_unb_ = 1.94, 95%CI = 0.64 to 2.35] and the short intermittent bout duration 3 × 4 min [peak HR: md = 5.12, *p* < 0,01 d_unb_ = 1.52, 95%CI = 1.53 to 8.71; %HR: md = 2.72, *p* < 0.01, d_unb_ = 1.1, 95%CI = 0.83 to 4.16; RPE: md = 0.87, *p* < 0.05, d_unb_ = 0.81, 95%CI = 0.02 to 1.72]. The full set of post-hoc pairwise comparisons are presented in Fig. 2.

### Technical and tactical performances

A significant interaction existed between the bout duration and the gender for % successful passes (F_2.30_ = 43.43, *p* < 0.001, ηp^2^ = 0.59), % successful tackles (F_2,30_ = 18.39, *p* < 0.001, ηp^2^ = 0.38), % successful duels (F_2,30_ = 148.38, *p* < 0.001, ηp^2^ = 0.83) and, % ball loss (F_2,30_ = 118.59, *p* < 0.001, ηp^2^ = 0.79) Table 1. This result may imply that the effects of bout duration on these variables depend on the gender of participants. Post hoc analyses showed that in female soccer players, the percentage of successful passes, successful tackles, successful duels, and ball loss were higher during the short intermittent bout duration (3 × 4 min) compared to 1 × 12 min [% successful passes: md = 2.62, *p* < 0.01, d_unb_ = 1.63, 95%CI = 1.07 to 4.17; % successful tackles: md = 18.7, *p* < 0.001, d_unb_ = 2.38, 95%CI = 12.83 to 24.68; % successful duels: md = 34.54, *p* < 0.001, d_unb_ = 3.14, 95%CI = 30.16 to 38.92; % ball loss: md = 3.81, *p* < 0.001, d_unb_ = 4.14, 95%CI = 3.02 to 4.59], and also during the medium intermittent bout duration (2 × 6 min) compared to 1 × 12 min [% successful passes: md = 1.38, *p* < 0.01, d_unb_ = 1.15, 95%CI = 0.25 to 2.5; % successful tackles: md = 13.8, *p* < 0.001, d_unb_ = 1.7, 95%CI = 5.97 to 20.39; % successful duels: md = 32.84, *p* < 0.001, d_unb_ = 6.3, 95%CI = 27.34 to 38.35; % ball loss: md = 3.62, *p* < 0.001, d_unb_ = 4.02, 95%CI = 3.02 to 4.59]. whereas, in soccer male players, the percentage of successful passes and tackles were higher during the continuous bout duration (1 × 12 min) compared to the medium intermittent bout duration (2 × 6 min) [ % successful passes: md = 2.91, *p* < 0.001, d_unb_ = 2.74, 95%CI = 1.97 to 3.85; %successful tackles: md = 8.8, *p* < 0.05, d_unb_ = 0.87, 95%CI = -0.09 to 17.7] and the short intermittent bout duration (3 × 4 min) [% successful passes: md 3.4, *p* < 0.001, d_unb_ = 2.31, 95%CI = 2.17 to 4.9; % successful tackles: md = 9.08, *p* < 0.05, d_unb_ = 0.9, 95%CI = -0.11 to 18.27]. However, 1 × 12 min was characterized by a lower % of ball loss compared to 2 × 6 min [ md = -2.34, *p* < 0.01, d_unb_ = 2.22, 95%CI = -3.48 to -1.39] and to 3 × 4 min [md = -2.12, *p* < 0.001, d_unb_ = -2.25, 95%CI = -2.97 to -1.27]. No significant difference was observed in % of successful duels within the different bout duration. The full set of post-hoc pairwise comparisons are presented in Table 1.

**Table 1:** Results of the mixed ANOVA with 3 × 2 repeated measures (Bout duration (1 × 12 min, 2 × 6 min and 3 × 4 min) x gender (female and male soccer players)

Main effects									
Variables	Bout duration			Gender			Interaction		
	F(_2,30_)	ηp^2^	Rating	F(_2,30_)	ηp^2^	Rating	F(_2,30_)	ηp^2^	Rating
Paces score	25.64***	0.46	Large	3.65	0.1	Medium	294.42***	0.9	Large
Peak HR of the game	27.03***	0.47	Large	69.42***	0.69	Large	5.27*	0.15	Large
% HR peak	27.27***	0.47	Large	15.55***	0.43	Large	5.38*	0.15	Large
La	28.65***	0.48	Large	3.33	0.1	Medium	21.69***	0.4	Large
RPE	36.84***	0.55	Large	65.57***	0.68	Large	4.9**	0.14	Medium
% of successful passes	2.58	0.07	Medium	0.33*	0.01	Small	43.43***	0.59	Large
% of successful tackles	2.04	0.06	Small	0.37	0.01	Small	18.93***	0.38	Large
% of successful duels	83.73***	0.73	Large	57.07***	0.65	Large	148.38***	0.83	Large
% of ball loss	7.42**	0.19	Large	18.62***	0.38	Large	118.59***	0.79	Large

PACES: Physical Activity Enjoyment Scale, *: *P* < 0.05; **: *P* < 0.01; ***: *P* < 0.001

## Discussion

The present study analyzed the difference in perceived enjoyment, physiological responses, RPE, and technical tactical performances within different bout duration: continuous bout duration (1 × 12 min), medium intermittent bout duration (2 × 6 min), and short intermittent bout duration (3 × 4 min), and between female and male soccer players during 4vs4 SSGs. The analyses revealed that both the bout duration and the gender affected the perceived enjoyment, the physiological responses, RPE, and the technical-tactical performance of female and male soccer players. Indeed, our results showed that these factors varied over the duration of SSGs, and according to the gender. Given the importance of training load (physiological responses, psychological stimulus, and technical tactical performance) in inducing training adaptations. Numerous studies [37] have examined how to manipulate the intensity of SSGs to use this specific exercise during physical training to achieve intensities sufficient to improve aerobic fitness [38, 39].

The major finding of this investigation was that a short intermittent bout duration (3 × 4 min), and the medium intermittent bout duration (2 × 6 min) have a positive influence on perceived enjoyment, peak HR, %HR, and technical tactical performance in female soccer players, On the other hand, in male soccer players these parameters were higher during the longer bout duration (1 × 12 min) except the ball loss (significant greater in 2 × 6 and 3 × 4 min) and successful duel (no significant difference within bout duration). Concerning La and RPE, the continuous bout duration (1 × 12 min) was characterized by higher values for both female and male soccer players.

The findings implied that in order to achieve higher levels of perceived enjoyment, physiological stimulus, and technical-tactical performance in the short term, physical trainers should take into account the gender and the duration of SSGs when planning SSGs training and that short and medium bouts should be selected over continuous bout for female soccer players, as opposed to that, one longer bout should be chosen over many intermittent bouts for male soccer players.

To the authors’ knowledge, this is the first study examining the relationship between perceived enjoyment and the length of SSGs bout in female and male soccer players. Our finding concerning the perceived enjoyment, quantified by the physical activity enjoyment scale (PACES) showed that female soccer players had a higher level of intrinsic motivation during the intermittent medium bout duration of SSGs than the continuous bout duration. This increase in perceived enjoyment could be explained by their satisfaction provides the essential ingredient for self-determined motivation during intermittent SSGs [7, 9, 11]. Furthermore, the players can be confronted with their own levels according to their capacities to improve their performance. If there are too many players on a playing field and the playing time is more extended, it causes negative effects on the pleasure of playing, limits the understanding and progress of the players in the game [40]. Indeed, they play football first for having fun, they want to run, touch the ball and participate in the game. Therefore, the environment in which players are placed during practice should aim to promote all of these desires [41]. In support, Köklü et al., [1] Gréhaigne et al., [40] and Zerai et al. [42] showed a greater variability in effort between players during SSGs than during controlled physical load exercises, this means that during SSGs some players will put in a lot of effort than others and it is more difficult to control the real efforts made by each player in SSGs. These variabilities of subjective causes are the origin of their inability to maintain a higher workload for a longer time. Moreover, the high rate of peripheral and mental fatigue following a long concentration to succeed in the tasks requested by the trainer decreases the pleasure of playing and maintaining performance [41]. For these reasons, female soccer players prefer to have a reduced recovery time between bouts duration in order to find more significant circulatory benefits than men [43, 44]. However, in male soccer players, the results were particularly noteworthy in terms of enhanced felt enjoyment when the bout duration was the longest. This could be explained by the fact that the continuous bout duration of SSGs resembles real football, which may motivate players to strive to achieve and experience greater feelings of competence [11]. In addition, the psychological need satisfaction provides the essential ingredient for their self-determined motivation [7, 19] and the positive emotion of enjoyment has been shown to be an important ingredient of motivation in elite male soccer players [45]. Another advantage of these types of game is that players significantly perceive these SSGs as less difficult and less boring than traditional races, despite almost identical cardiovascular responses [40].

Concerning physiological responses, our study results demonstrate that SSGs bout duration influences peak HR, %HRpeak, La, and RPE in female and male soccer players. Indeed, the peak of HR, and the % HR were significantly higher during the continuous and, medium bout duration than the short bout duration in female soccer players, whereas, in male soccer players, these values were significantly higher only during the continuous bout duration. Values of La and RPE were significantly higher during the continuous bout duration in female and male soccer players. Previous studies [1, 19, 46, 47], showed that variability in bout duration changes physiological reactions and game behavior in soccer players, which coincides with our results. It seems that the medium and the continuous bout duration are effective in producing work intensities compatible with the development of maximum aerobic power in female and male soccer players [40]. In line with our hypothesis, the continuous bout duration induced an intensity of ~ 90% of peak HR that is similar to the intensity achieved in a soccer match [19, 46, 48]. These findings are thought that a continuous bout duration is causing to be more aerobic as compared to the short intermittent bout duration SSGs [1] and, these values are considered to be an adequate stimulus for the cardiovascular system [1, 6, 10].

In the same context, [49] suggested that continuous SSGs stimulate the cardiovascular system in both genders, as HRpeak and %HR of players in this study were over 90% of individual HRpeak. In female soccer players, our results have shown that the medium intermittent bout duration also increases HR responses. The possible reason for this finding is that players have a positive enjoyment when they could perform more technical actions such as a number of ball contact, duel, and tackle in medium bout durations than short bout duration in SSGs.

Although previous studies have examined the exercise intensity of SSGs [50], no investigations have verified the effect of gender and the length of bout duration on technical actions. knowing that SSGs are commonly used as a training modality for involving both physical and technical components and that several physical trainers use SSGs especially for technical purposes [39], the examination of the effect of gender, and the bout duration on technical actions are determining. Our results revealed that female players seemed to be more technically proficient during continuous and medium intermittent bouts than short intermittent bouts, while male soccer players seemed more technically only during the continuous bouts. Indeed, % successful passes, % successful tackles, % successful duels, and % successful ball loss were enhanced during the continuous and the medium intermittent bouts compared to the short intermittent bout in female soccer players, as a result in male soccer players, the % of successful passes and successful tackles were enhanced during the continuous bout compared to the intermittent bout duration of SSGs (2 × 6 min and 3 × 4 min) and, the % of ball loss was higher during the intermittent but duration. This may be explained by the increased perceived enjoyment, which may translate into better technical-tactical efficiency. This reasoning is consistent with the results of previous studies measuring enjoyment and mood responses to SSG [9, 11, 19]. On the specific influence of the gender of players on the technical-tactical performance, it is worth highlighting that all variables of technical-tactical performance were obtained in female soccer players during the intermittent formats compared to male soccer players, where they obtained better % of successful passes, and tackles during the continuous bout duration. These results may be explained by the effect of peripheral fatigue in female soccer players during longer sessions of SSGs (in our studies higher lactate and RPE values compared to male soccer players) and even by the influence of concentration and motivation of coaches to succeed in technical gestures during games. Indeed, in SSGs, these characteristics are found because players can be confronted with their own levels according to their age and their abilities to improve their performance. If there are too many players on a playing field, this has negative effects in the success of technical gestures, limits the understanding and progress of the players in the game. Also, the environment in which the players are placed during the practice should aim to promote all of these desires [40].

## Conclusions

In conclusion, this study demonstrated that the duration of small-sided games (SSGs) and the gender of players had significant effects on perceived enjoyment, physiological responses, and technical-tactical performance in soccer players. Female players showed higher levels of enjoyment and technical-tactical performance during medium and short intermittent bout durations, while male players exhibited these improvements during a longer continuous bout duration. These findings suggest that coaches and trainers should consider the gender and bout duration when designing SSGs-based training programs to optimize perceived enjoyment, technical-tactical competence, and training load, as indicated by higher heart rate peaks and lactate concentrations. By tailoring SSGs accordingly, coaches can enhance the effectiveness and engagement of training sessions for soccer players.

### Practical applications

This study showed that SSGs bout duration and the gender of players influenced perceived enjoyment, physiological responses, and technical-tactical performance of soccer players. Female soccer players favor a 2 × 6 min and 3 × 4 mi interval-type format over 1 × 12 min continuous bout duration, however, male soccer players favor 1 × 12 min continuous bout duration over 2 × 6 min or 3 × 4 min interval-type formats. From a practical point of view, coaches should consider gender when programming SSGs-based training to significantly increase perceived enjoyment, technical-tactical competence, and training load, due to provoking higher heart rate peak and lactate concentrations.

## Data Availability

The data that support the findings of this study are openly available upon request from the corresponding author.
